# Culture of Mesenchymal Stem Cells Derived From the Infrapatellar Fat Pad Without Enzyme and Preliminary Study on the Repair of Articular Cartilage Defects in Rabbits

**DOI:** 10.3389/fbioe.2022.889306

**Published:** 2022-08-17

**Authors:** Qiwei Fu, Rong Zhou, Jia Cao, Yi Chen, Jun Zhu, Yiqin Zhou, Jiahua Shao, Wei Xin, Shuai Yuan

**Affiliations:** ^1^ Department of Orthopedics, Shanghai Changzheng Hospital, Naval Medical University, Shanghai, China; ^2^ Department of Orthopedics, 72nd Group Army Hospital of the PLA, Huzhou City, China

**Keywords:** cartilage damage, repair, mesenchymal stem cells, infrapatellar fat pad, enzymatic digestion introduce

## Abstract

**Objective:** The aim of the study was to evaluate the advantages of without enzyme isolating patellar fat pad-derived mesenchymal stem cells (IPFP-SCs) and the feasibility of cartilage repair.

**Methods:** The IPFP-SCs were isolated using the without enzyme method and compared with the IPFP-SCs obtained by the traditional enzyme digestion method in terms of cell proliferation ability, characterization, and differentiation ability, and the differences in chondrogenic induction and differentiation between the two groups were compared. Twenty-four New Zealand rabbits were randomly divided into four groups (*n* = 6). After the articular cartilage defects were modeled, different preparations were injected into the joint cavity. The rabbits in the group A were injected with the mixture of IPFP-SCs and pure PRP (P-PRP), separated using the without enzyme method, while those in the group B were injected with the mixture of IPFP-SCs and P-PRP separated with the digestion method, while those in the group C were injected with SVF separated using the without enzyme method, and those in the group D were injected with normal saline. At 6 weeks and 12 weeks after operation, the cartilage repair of rabbit joint specimens was observed and evaluated by gross observation and histological staining, and the effects of different IPFP-SCs application forms in repairing cartilage defects were compared.

**Results:** The time required to obtain IPFP-SCs by enzyme-free isolation was significantly less than that by enzyme digestion, while the acquisition rate of primary cells was significantly lower than that by enzyme digestion. After culture and amplification, the two IPFP-SCs from different sources did not show significant differences in cell proliferation, cell phenotype, and differentiation ability. In animal experiments, groups A and B had the best effect on the repair of cartilage defects, and there was no significant difference between the two groups. The repair effect in group C was weaker than that in the former two groups, but it was relatively better than that in group D.

**Conclusion:** It is more time-saving to obtain IPFP-SCs by the without enzyme method than by enzymatic digestion, and there is no significant difference in cell identification and differentiation potential between the two methods. However, the rate of obtaining primary cells was significantly lower than that with the enzyme digestion method. IPFP-SCs showed good repair effect in the rabbit animal cartilage defect model, providing ideas and reference for the clinical application of stem cells in repairing articular cartilage.

## 1 Introduction

Patients with osteoarthritis (OA) are very common in clinical practice, with well-defined joint pain and limited mobility, yet the treatment has been a difficult challenge. The reason is that the articular cartilage structure is highly differentiated, no blood vessels, and no nerves, which makes the articular cartilage lack the ability to regenerate and heal itself, and once injured, even small defects are difficult to get repaired by themselves (Alford and Cole, 2005). The development of regenerative medicine has brought hope for the treatment of OA. The clinical trials of stem cell therapy for articular cartilage regeneration have been carried out in some countries and regions. Adipose-derived stem cells (ADSCs) for cartilage repair and treatment of OA have also received widespread scholarly attention (Bateman et al., 2018). The main forms of application of ADSCs for cartilage repair are two types: one is in the form of stromal vascular fraction (SVF), and the other is culture-expanded ADSCs. Most of which provide ADSCs in the form of SVF (Liu et al., 2014; Vonk et al., 2015). In addition to ADSCs, SVF also contains endothelial cells, immune cells, pericytes, and other stromal components, and ADSCs can be obtained by plastic attachment followed by culture and expansion after isolation of SVF.

ADSCs have shown promising application in clinical and basic research results, but there is no uniform standard regarding the protocol of tissue isolation and cell extraction. The traditional method for isolating ADSCs is a tissue digestion method, which relies on collagenase of bacteria origin, and the quality of collagenase with different batches and suppliers may be a variable factor, producing different digestion results (Williams et al., 1995; Zuk et al., 2001; Zuk et al., 2002). In order to improve the quality of ADSCs isolation while reducing the impact of cost, time, and other factors, in recent years, researchers have become interested in alternative methods for extracting ADSCs (De Francesco et al., 2018). It has been proposed that the without enzyme method can be used to isolate and obtain ADSCs, especially when dealing with large amounts of fat aspirates for autologous use (Shah et al., 2013). Treatment of emulsified adipose tissue using without enzyme digestion can isolate SVF containing large amounts of ADSCs, and instruments for SVF extraction using without enzyme methods are already in clinical practice.

Subcutaneous tissue and subpatellar adipose tissue are two main sources of ADSCs. The properties and capacity of subpatellar fat pad-derived stem cells (IPFP-SCs), as one of ADSCs, have received much attention (do Amaral et al., 2017). An experimental study comparing knee-derived IPFP-SCs from OA knee and cruciate ligament reconstruction surgery patients showed similar chondrogenic potential, which provides confidence for future clinical applications using autologous IPFP-SCs from OA patients and suggests that IPFP-SCs are a good source of stem cells for cartilage repair (Liu et al., 2014). The repair effect of ADSCs alone on injured cartilage is limited, and the strategy of finding suitable deliverables or adjuvants to be used in combination with ADSCs is also a hot research topic (Koh et al., 2014). Growth factors can enhance the proliferation and chondrogenic capability of ADSCs. In fact, PRP (platelet-rich plasma) has been used in clinical trials as a growth factor-rich source in combination with ADSCs (Pak et al., 2016). PRP can promote the deposition of proteoglycan and the production of type II collagen in IPFP-SCs chondral pellets. The white blood cell-free P-PRP (pure PRP) is superior to the white blood cell-containing L-PRP (PRP-containing Leucocytes) in promoting chondrogenic differentiation.

In this study, the IPFP-SC was isolated, extracted and isolated without enzyme, and compared with the IPFP-SC obtained by the traditional enzyme digestion method in terms of cell proliferation, characterization, and differentiation. At the same time, the rabbit model of articular cartilage defect was established using intra-articular injection for an *in vivo* experimental study, to evaluate the cartilage defect repair ability of IPFP-SCs obtained with different methods combined with PRP.

## 2 Research Methods

### 2.1 Experimental Grouping

A total of 24 healthy New Zealand rabbits, all male, 4 months old, weighing 3–3.5 kg, were used for the experiment. The remaining 24 rabbits were randomly divided into four groups (*n* = 6, 12 knees in each group). Group A: articular cavity injection of IPFP-SC + P-PRP separated by the without enzyme method to repair cartilage defects. Group B: IPFP-SCs + P-PRP separated by intra-articular injection and digestion to repair cartilage defects. Group C: SVF separated by the without enzyme method by intra-articular injection to repair cartilage defects. Group D: normal saline was injected into articular cavity.

### 2.2 Isolation and Culture of IPFP-SCs

#### 2.2.1 Without Enzyme Method

After adequate skin preparation and disinfection, an anteriormedium incision was made in the rabbit’s right knee joint to expose the patella. Using the medial parapatellar approach, the patella was laterally dislocated to expose the distal femur’s articular surface. The IPFP was obtained and dissected behind the patellar ligament with sterile ophthalmic scissors. Approximately 1 cm of IPFP tissue was collected and finely minced using the scissors. After dilution with 5 ml of PBS solution and re-suspension by repeated pipetting for half a minute, and centrifuged at 260 g for 5 min. The tissue fluid mainly composed of adipose tissues at the upper part was discarded, and the histiocyte mass at the bottom of the centrifuge tube cone was retained to obtain SVF by the without enzyme method. The SVF was re-suspended in DMEM/F12 medium (1:1 volume ratio of DMEM to F12) with 10% FBS, 1% PS, seeded into culture vessels, and placed in a thermostatic cell incubator for cell culture (culture environment 37°C, 5% CO_2_). The attachment of primary cells P0 was observed and the medium was replaced on the third day, and then the medium was replaced every 3 days. When the primary cells in the culture flask were distributed in clusters, grew well, and fused by more than 60%, digestion and passage were performed.

#### 2.2.2 Enzyme Digestion Method

The rabbit infrapatellar adipose tissue was cut and crushed into a paste with the particle diameter less than 1 mm^3^, 0.1% type I collagenase solution was added to digest adipose tissue, with adipose tissue volume to digestive juice volume ratio of 1:3–1:4. The samples were placed in a thermostatic oscillation box, after 60 min at 37°C and 150 r/min, DMEM medium solution was added to stop digestion. After filtration, the samples were centrifuged at 260 g for 5 min, the upper layer of digestive juice was discarded, 2 ml of red blood cell lysate was added for re-suspension, standing for 5 min, followed by 2 ml of PBS, and then centrifuged again at 260 g for 5 min to remove the supernatant, and the SVF by the digestion method was obtained at the bottom of the centrifuge tube. The digestion passage operation was the same as the without enzyme method.

### 2.3 Establishment of Rabbit Knee Joint Cartilage Defect Model

The diet and drinking water of experimental rabbits were stopped 12 h before surgery. Intravenous general anesthesia was administered by intravenous injection at the ear margin with 3% sodium, and the drug dose was calculated as 30 mg/kg. Backup skin with onset of anesthesia, supine position, four limbs with restraint. The incision approach was the anterior medial knee approach, about 3 cm in length, and the incision was made layer-by-layer. The patella was dislocated laterally through the medial aspect of the patella to expose the knee joint. After exposing the trochlea of the distal femur, a cylindrical full-thickness cartilage defect (4 mm in diameter and 1.5 mm in depth) was induced on the trochlear groove using a sterile hand drill with the knee in full flexion. After irrigation of the surgical field, the joint capsule and the deep fascia were tightly sutured. The intra-articular injection was performed through the deep fascia layer joint puncture and slow bolus injection. The subcutaneous and skin of the incision was intermittently sutured.

Group A: IPFP-SCs were isolated and extracted by the without enzyme method. The rabbit peripheral blood was collected, and P-PRP was collected using two centrifugations. A 10% volume of sterile 228 mM CaCl_2_ solution was added before use, and 0.5 ml was withdrawn for the experiment. After the bilateral knee joint modeling was established, 0.5 ml cell suspension +0.5 ml P-PRP was injected into each knee joint cavity through the deep fascia layer. After closure of the incision, the dressing was covered with bandage. The rabbits were given intramuscular injection of penicillin sodium 10 WU into the buttock to prevent infection, the ears of the rabbits were marked without joint fixation, and the experimental rabbits were returned to the rabbit cage. After the experimental rabbits awoke, vital signs, wound bleeding, and healing were observed. Intramuscular penicillin sodium was given at a dose of 50,000 IU/kg per day for 3 days after surgery.

Group B: IPFP-SCs were isolated and extracted by digestion. The closure of the incision and subsequent procedures are the same as those in group A.

Group C: the single inferior adipose tissue was cut off, cut into pieces, re-suspended, without enzyme digestion, and centrifuged to collect SVF cell clusters, and re-suspended with 1 ml normal saline. After the experimental rabbits knee joint modeling operation was completed, l ml of without enzyme SVF was injected into each knee joint cavity through the deep fascia layer. The closure of the incision and subsequent procedures are the same as those in group A.

Group D: blank control group. After the cartilage defect model was established, the operation field was irrigated and 1 ml of normal saline was injected into the joint cavity. The closure of the incision and subsequent procedures are the same as those in group A.

### 2.4 Observation Indicators

#### 2.4.1 Primary Cell Colony Formation Experiment

SVF obtained by two pathways of the without enzyme method and enzyme digestion method. Three aliquots of each tissue sample were inoculated into 6-well plates, 2 ml DMEM/F12 medium was added to each well, and primary cell culture was performed in a 37°C incubator containing 5% CO_2_. Afterward, fresh medium was changed every 3 days. After 14 days of inoculation, the medium was discarded and the wells were washed with PBS. Methanol solution was added and allowed to settle for 20 min. Crystal violet staining solution working solution was added into each well, and excess staining solution was removed after standing at room temperature for 20 min. Another three parallel experiments were performed for each group. The culture medium was discarded. After washing with PBS, 0.25% trypsin solution was added for reaction for 1 min. DMEM/F12 medium (0.5 ml) was added to terminate digestion. The cells were blown, and collected for counting using a cell counting plate.

#### 2.4.2 Cell Proliferation Experiment Using CCK-8 Method

IPFP-SCs cells isolated by the without enzyme and enzymatic digestion methods at passage P3 were collected and seeded in five 96-well plates, and the absorbance (OD value) at 450 nm was measured using a microplate reader at time points of 12, 24, 48, 72, and 96 h, respectively.

#### 2.4.3 Detection of Cellular Immunophenotype by Flow Cytometry

IPFP-SCS obtained by the without enzyme method and enzymatic digestion at P3-generation were collected, and monoclonal antibodies CD44, CD90, CD45, and CD34 (BD Pharmingen, United States) were selected for flow cytometry. The collected cells were re-suspended in PBS and incubated with fluorescently labeled monoclonal antibodies CD44-BB700, CD90-PE, CD45-FITC, CD34-Alex Flour 488, and Mouse IgG1-PE for 30 min in the dark. Unstained non-fluorescently labeled cells were isotype controls. The test results were processed using FlowJo 10 software.

#### 2.4.4 Comparison of Adipogenic and Osteogenic Differentiation Potentials of Two IPFP-SCs

IPFP-SCs obtained under good culture condition through P3-generation and without enzyme and digestion methods were selected. Adipogenic differentiation potential: Both IPFP-SCs were seeded into 6-well plates for culture. After culture for 3 days, the adipogenic differentiation medium was replaced when the degree of cell fusion reached 100%. Adipogenic differentiation induction solution was replaced every 3 days of induction, and differentiation was induced for 21 days for staining with oil red O staining solution. Staining procedure of oil red O staining solution: the induction solution was sucked off and rinsed with PBS twice, the cells with 4% paraformaldehyde solution was fixed for 30 min, rinsed with PBS twice, stained with 0.3% oil red O staining solution for 30 min, and rinsed with PBS twice. The adipogenic differentiation was observed under the microscope. Osteogenic differentiation potential: 0.1% gelatin was used to coat the bottom of each well of 6-well plates in advance. After collecting the two types of cells, they were seeded into 6-well plates for culture, and when the cell confluence reached 60%–70%, the osteogenic induction and differentiation culture medium was replaced. The osteogenic differentiation induction solution was replaced every 3 days. When obvious calcium nodules were observed, the medium was changed once every 2 days and induced to differentiate for 21 days, followed by alizarin red staining. Alizarin red staining procedure: the induction solution was sucked, rinsed with PBS for two times, fixed with 2 ml of 4% paraformaldehyde solution for 30 min, rinsed with PBS for two times, stained with 0.5% alizarin red solution for 30 min, the staining solution was sucked, and rinsed with PBS for two times. Osteogenic differentiation was observed under a microscope.

#### 2.4.5 Comparison of Chondrogenic Differentiation Potential of Two IPFP-SCs

The chondrogenic induction differentiation assay was performed by the crawling assay. The lysine-coated climbing tablets were previously placed in sterile 24-well plates, and the IPFP-SCs cells were re-suspended with chondrogenic differentiation induction solution, inoculated into 24-well plates. The cells in the two groups were isolated in three wells (1 ml induction solution for each well), and the fresh induction solution was changed once every 3 days. After 14 days of culture, immunofluorescence staining was performed. The immunofluorescence staining was observed under the laser confocal microscope.

#### 2.4.6 Histological Observation

At 6 and 12 weeks after operation, six knees of three rabbits in each group were euthanized by air embolism after intravenous anesthesia. The knee was incised along with an anterior knee incision, exposed, and freed, and the distal femur was cut with a motorized oscillating saw. The repair of the defect area of the joint specimen was macroscopically observed and preliminarily assessed. Joint specimens were first decalcified, embedded in paraffin, and sectioned sequentially. Gross observation and preliminary evaluation of joint specimen defect repair. The joint specimen was first decalcified, embedded in paraffin, and then sectioned in sequence. The gross and tissue section results of the specimen were scored according to Moran’s gross articular cartilage scoring criteria (eight points) and cartilage repair histological score (14 points) (Moran et al., 1992).

### 2.5 Statistical Analysis

Measurement data in the obtained results were expressed as “mean ± standard error.” The statistical software used was: GraphPad Prism 7. For comparison among multiple groups, if the data were subjected to normal distribution, one-way ANOVA test would be used for comparison. If the normal distribution is not true, the Kruskal–Wallis test is used. T-tests are used for pairwise comparison. The test levels of the aforementioned test methods were 0.05, and *p* < 0.05 indicated that the difference was statistically significant.

## 3 Result

### 3.1 Culture and Identification of IPFP-SCs

**FIGURE 1:**
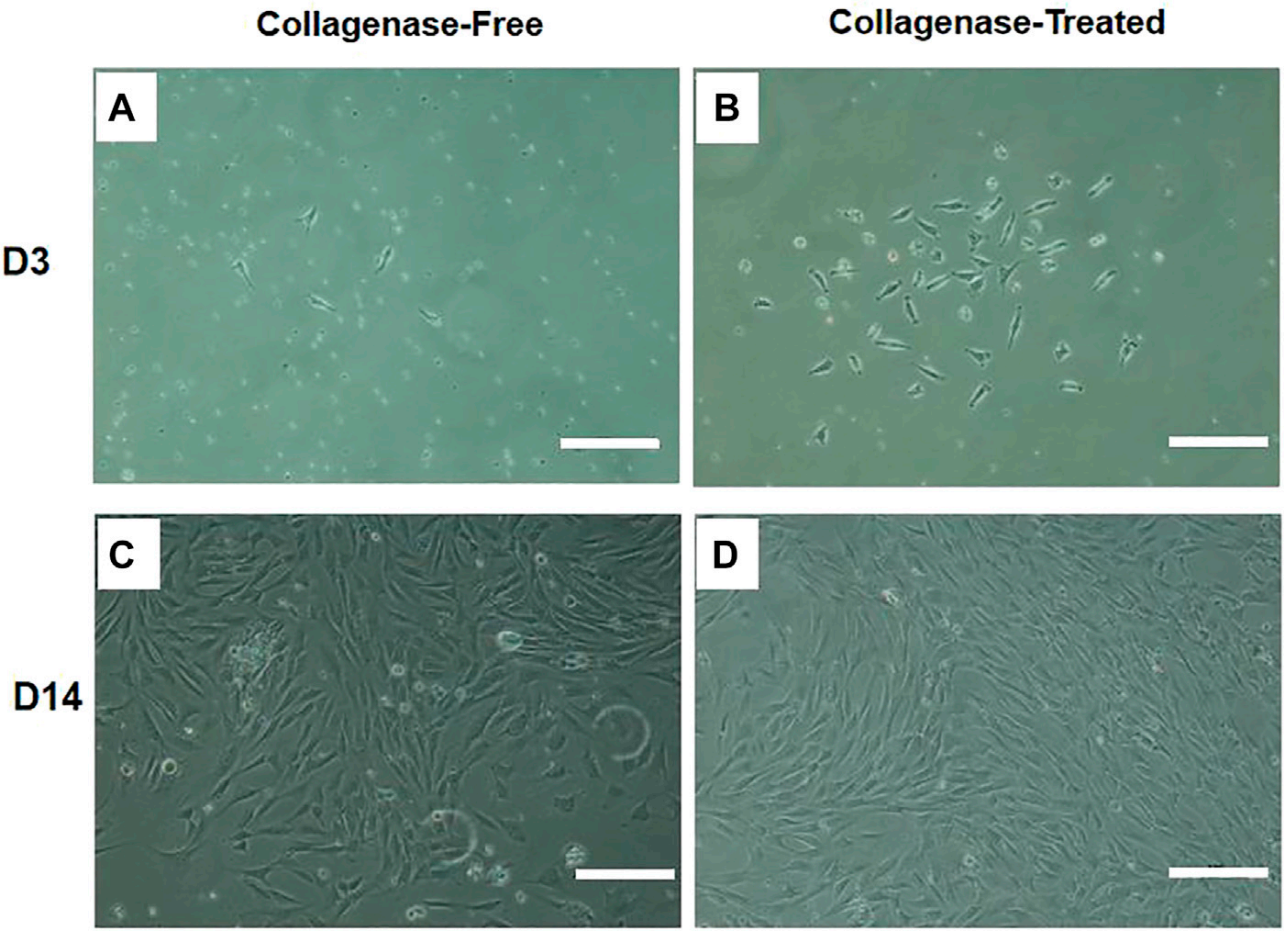
Representative diagram of IPFP-SCs extraction by the without enzyme method and digestion method. Scale bar = 200 μm.

**FIGURE 2:**
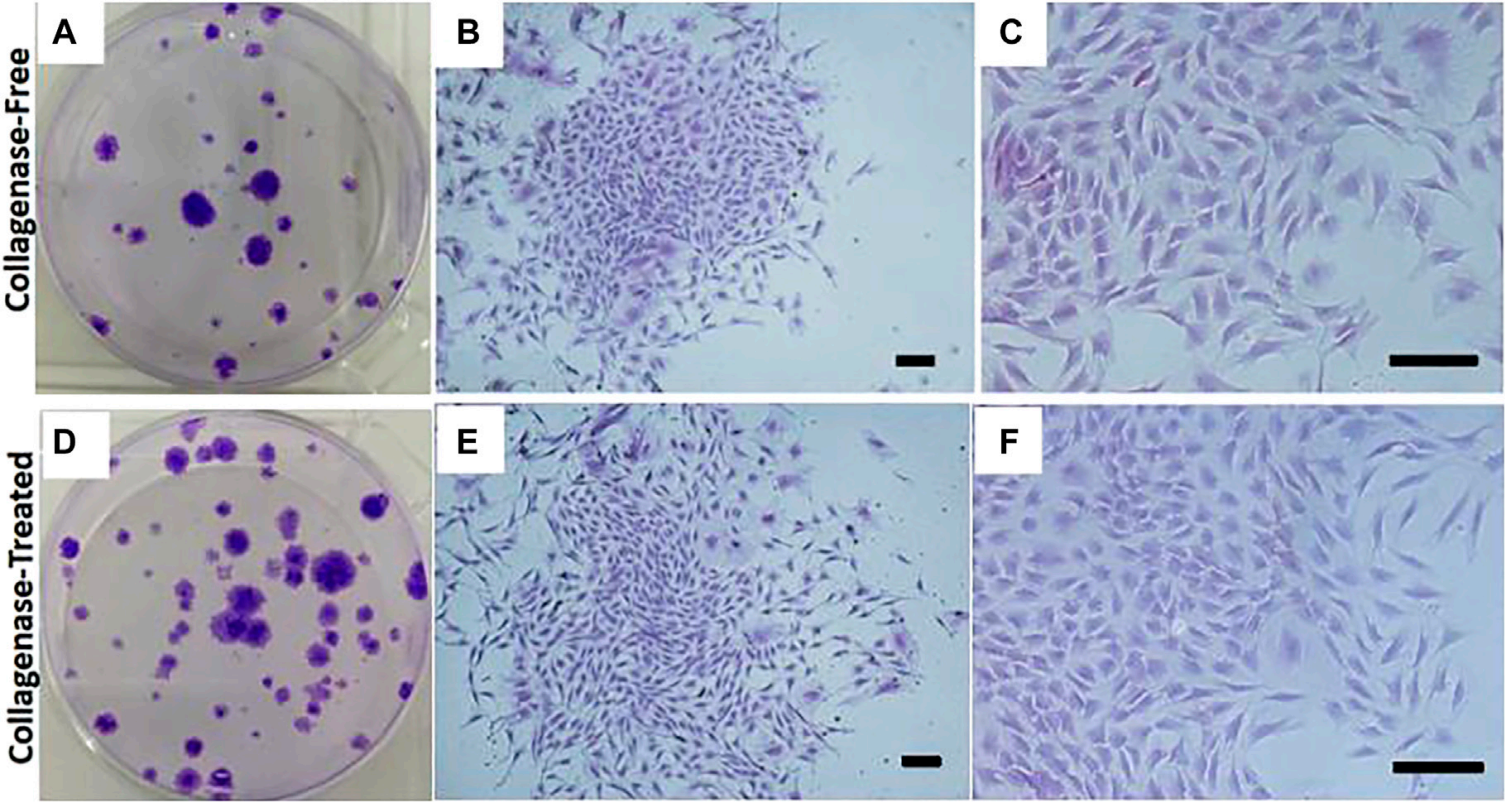
Representative colony assay of primary cell culture isolated and extracted by the without enzyme method and enzyme digestion method. Scale bar = 200 μm.

The attachment of fusiform cells was observed 48 h after inoculation with SVF isolated by both the without enzyme and enzyme digestion methods. The adherent cells observed using the without enzyme method were relatively “rare” (as shown in Figures 1A,B). The cells that grew in clusters could be observed after 14 days culture. The colonies in the enzyme-free group were significantly less than that in the enzyme digestion method. The cells were generally consistent in morphology as observed under the microscope, and the cells resembled a spindle, took the shape of a long spindle, and gathered in clusters (as shown in Figures 1C,D). After the primary cells were isolated in the culture plate for 14 days, the colony formation was observed after staining with crystal violet staining. The number of colonies formed by without enzyme method (Figure 2A) was significantly lower than that by the enzyme digestion method (Figure 2D). After staining with crystal violet, it was observed that the two cells were in general consistent with each other in morphology and grew in clusters (Figures 2B,C,E,F). After 14 days of culture, the acquisition rate of primary cells by the without enzyme method (3.17 ± 0.20)×10^5^/g was significantly lower than that by the enzyme digestion method (13.64 ± 0.84)×10^5^/g (*p* < 0.05).

### 3.2 Comparison of Cell Proliferation Abilities of IPFP-SCs

**FIGURE 3:**
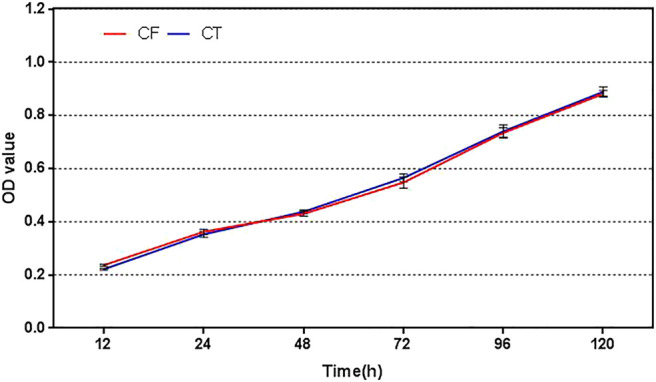
Cell proliferation profiles of cells obtained by the without enzyme method and enzyme digestion method. CF: collagenase free, without enzyme, CT: collagenase treated, enzyme digestion, and OD: absorbance.

The results of CCK-8 showed that the proliferation rates of the two cells were almost parallel. After 48 h, the OD value of the digestion group was slightly higher than that of the without enzyme group, and there was no statistical difference between the two groups (*p* > 0.05), as shown in Figure 3.

### 3.3 Identification of Cell Phenotype of IPFP-SCs

**FIGURE 4:**
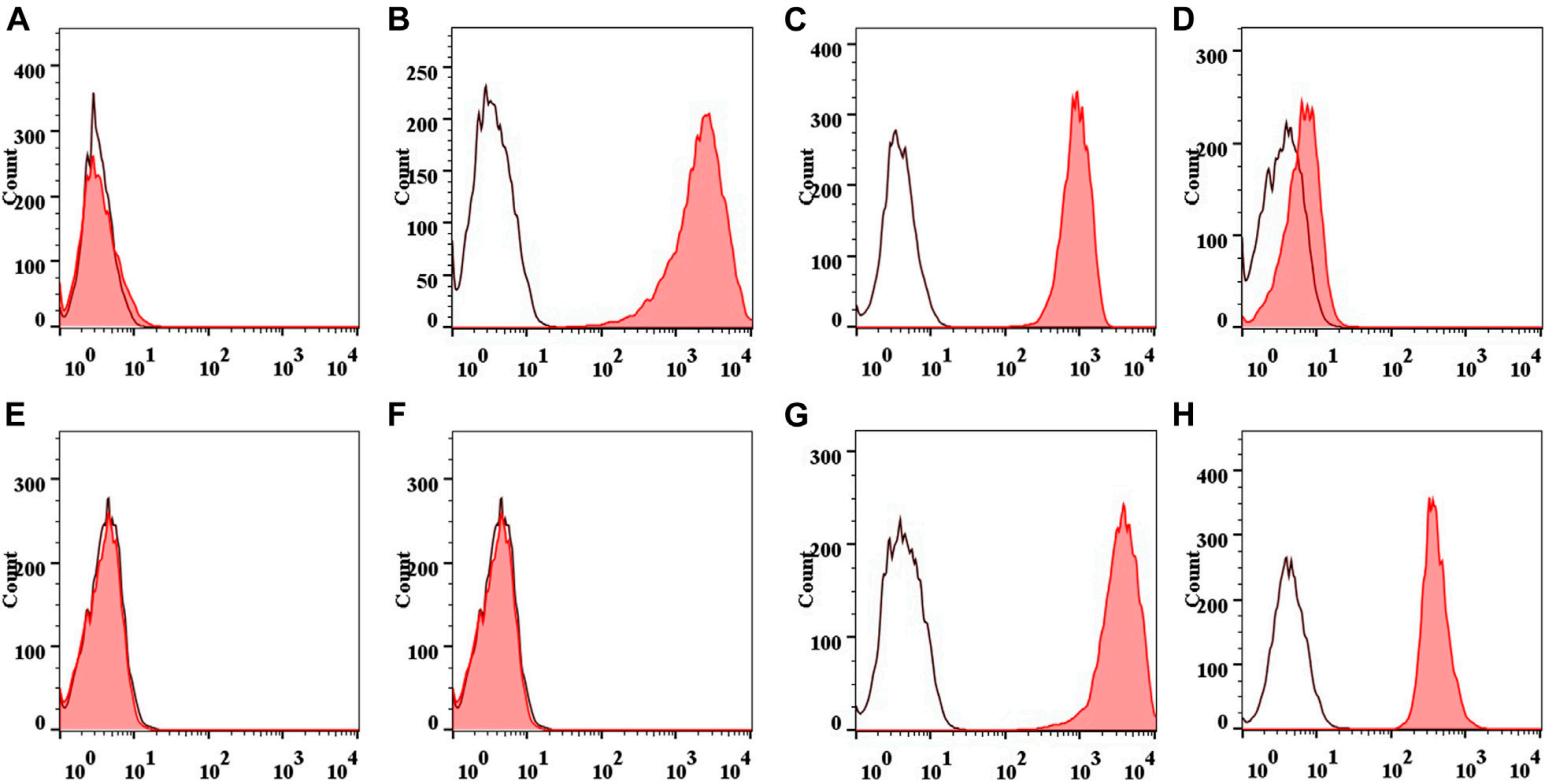
Flow diagram of IPFP-SCs surface markers. Collagenase free: CD44 **(A)**, CD90 **(B)**, CD45 **(C)**, and CD34 **(D)**. Collagenase treated: CD44 **(E)**, CD90 **(F)**, CD45 **(G)**, and CD34 **(H)**.

The positive rates of CD44 expression and CD90 expression were (98.89 ± 0.23) and (98.92 ± 0.25)%, (98.59 ± 0.27), and (98.54 ± 0.29)%, respectively. The positive rates of CD45 expression and CD34 expression were (0.95 ± 0.07) and (0.95 ± 0.04)%, (1.41 ± 0.11) and (1.69 ± 0.09)%, respectively. There was no statistical difference between the two groups with the same marker (*p* > 0.05). Typical flow results are shown in Figure 4.

### 3.4 Lipogenic and Osteogenic Differentiation Potential of IPFP-SCs

**FIGURE 5:**
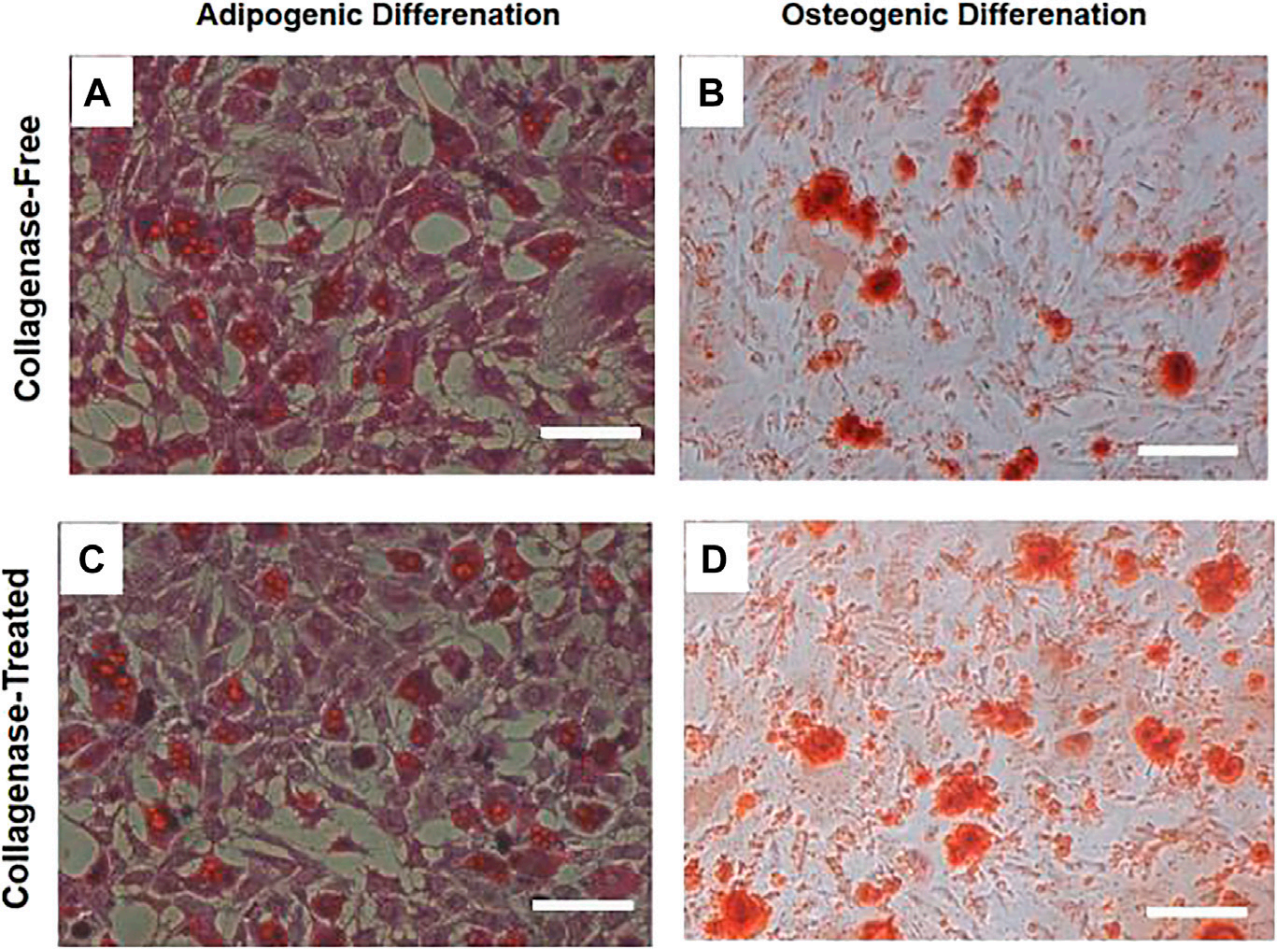
Staining diagram of lipogenic and osteogenic induction and differentiation of two IPFP-SCs obtained by the without enzyme method and digestion method. After adipogenic induction and differentiation of the two cells, they were treated with oil red. On staining, fused lipid droplets **(A,C)** could be observed, osteogenic-induced alizarin red staining could be observed, fused calcium nodules **(B,D)** could be observed, and relatively many images of calcium nodules could be seen in the results of enzymatic digestion sources. Scale bar = 200 μm.

To verify the adipogenic and osteogenic differentiation ability of IPFP-SCs isolated and extracted using the without enzyme method, adipogenic-induced cells were stained with oil red O, and osteogenic-induced cells were stained with alizarin red. The cells derived from the two methods could differentiate into adipocytes and deposited mineralized tissue indicative of osteoblast differentiation, as shown in Figure 5.

### 3.5 Chondrogenic Differentiation Potential of IPFP-SCs

**FIGURE 6:**
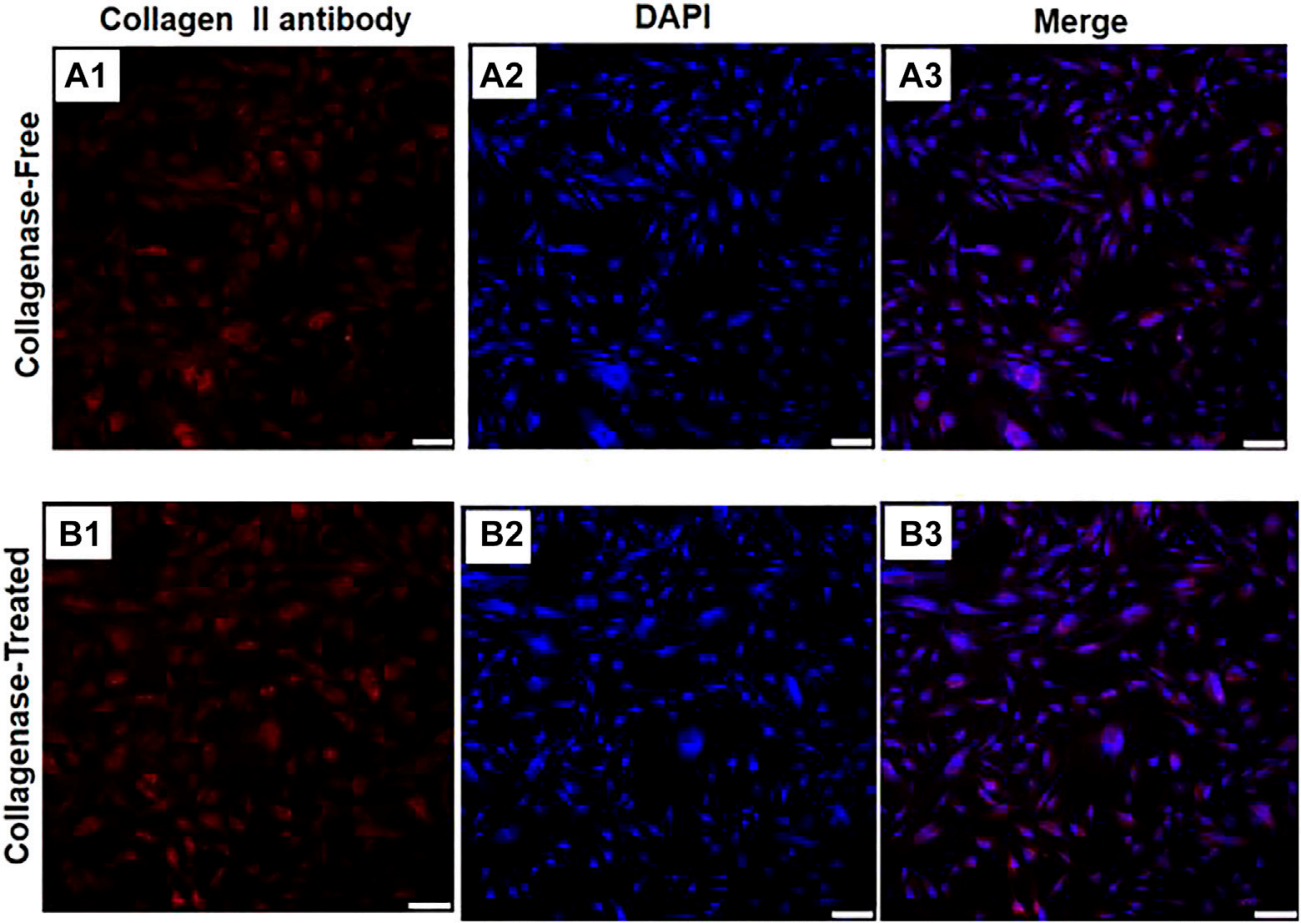
Immunofluorescence diagrams of two IPFP-SCs obtained using the without enzyme method and digestion method after chondrogenic induction. **A1** type II collagen produced by the cell matrix after chondrogenic induction of IPFP-SCs using the without enzyme method is positively expressed; type II collagen produced by the cell matrix after chondrogenic induction of IPFP-SCs with **B1** enzyme digestion was positively expressed, and all of them showed orange red color. **A2** and **B2** were nuclear images stained with DAPI, and the nuclei were blue. **A3** and **B3** were combined images of the corresponding cells. Scale bar = 100 μm.

For the cells isolated by the without enzyme method and with enzyme digestion method, after undergoing chondrogenic induction and differentiation into chondrocytes, positive expression of type II collagen (orange–red cell matrix) could be seen without significant difference, as shown in Figure 6.

### 3.6 Comparison of Rabbit Joint Observation

**FIGURE 7:**
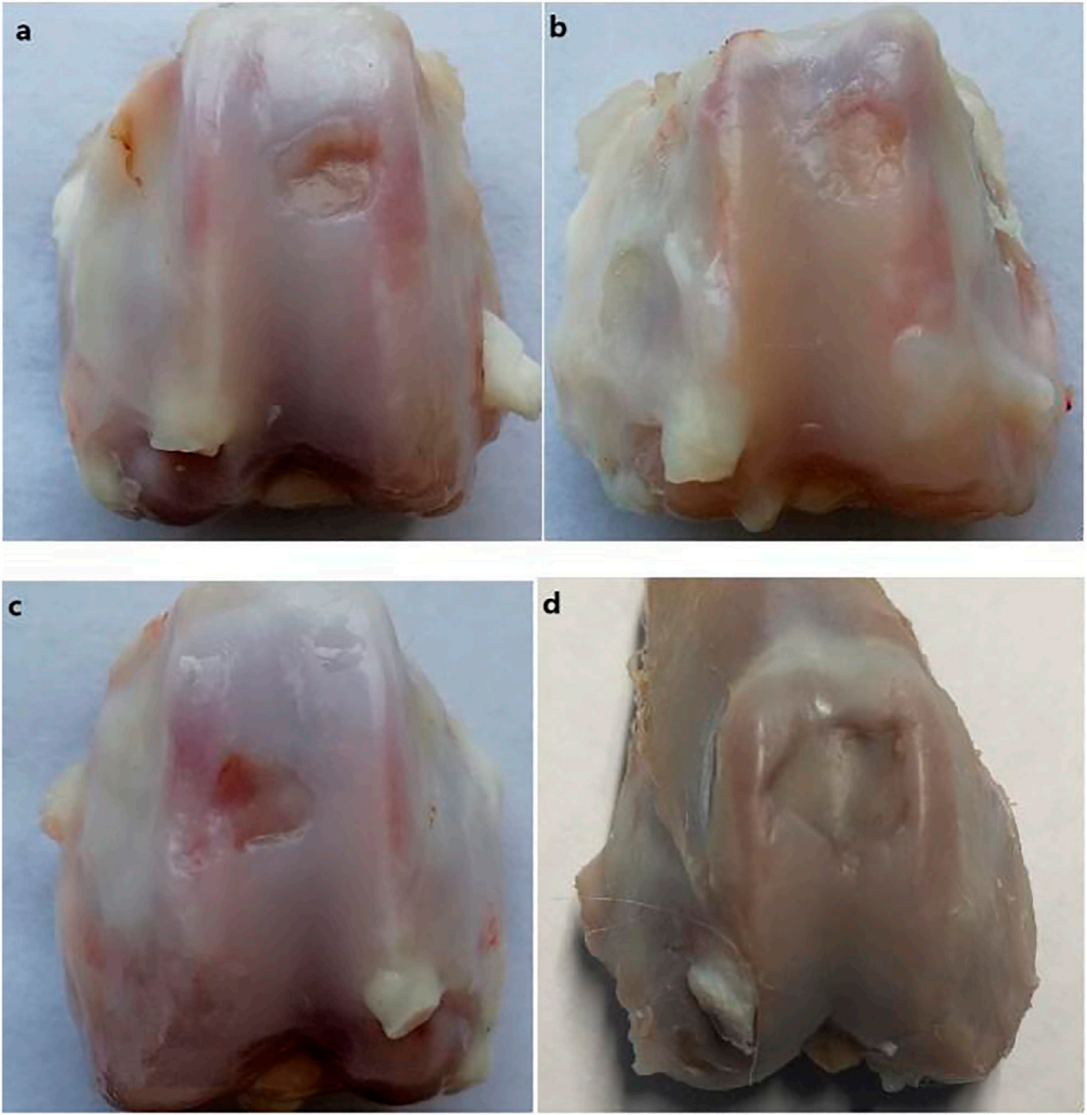
Gross views of four groups of specimens taken at 6 weeks after surgery.

The gross observations of the joint specimen, 6 weeks after surgery are shown in Figure 7. Group A: the cartilage defect area was filled with new tissue, with pitting, unevenness, and clear boundary (Figure 7A). Group B: the cartilage defect area was filled with new tissues, with depressions, unevenness, and clear boundaries (Figure 7B). Group C: the new tissue was found in the cartilage defect area, which was thinner than those in Groups A and B, with uneven surface, clear boundary of the depression, and damaged cartilage tissue near the medial margin (Figure 7C). Group D: the defect area was depressed with clear boundaries, and no significant new tissue formation and peripheral cartilage tissue damage was observed (Figure 7D).

**FIGURE 8:**
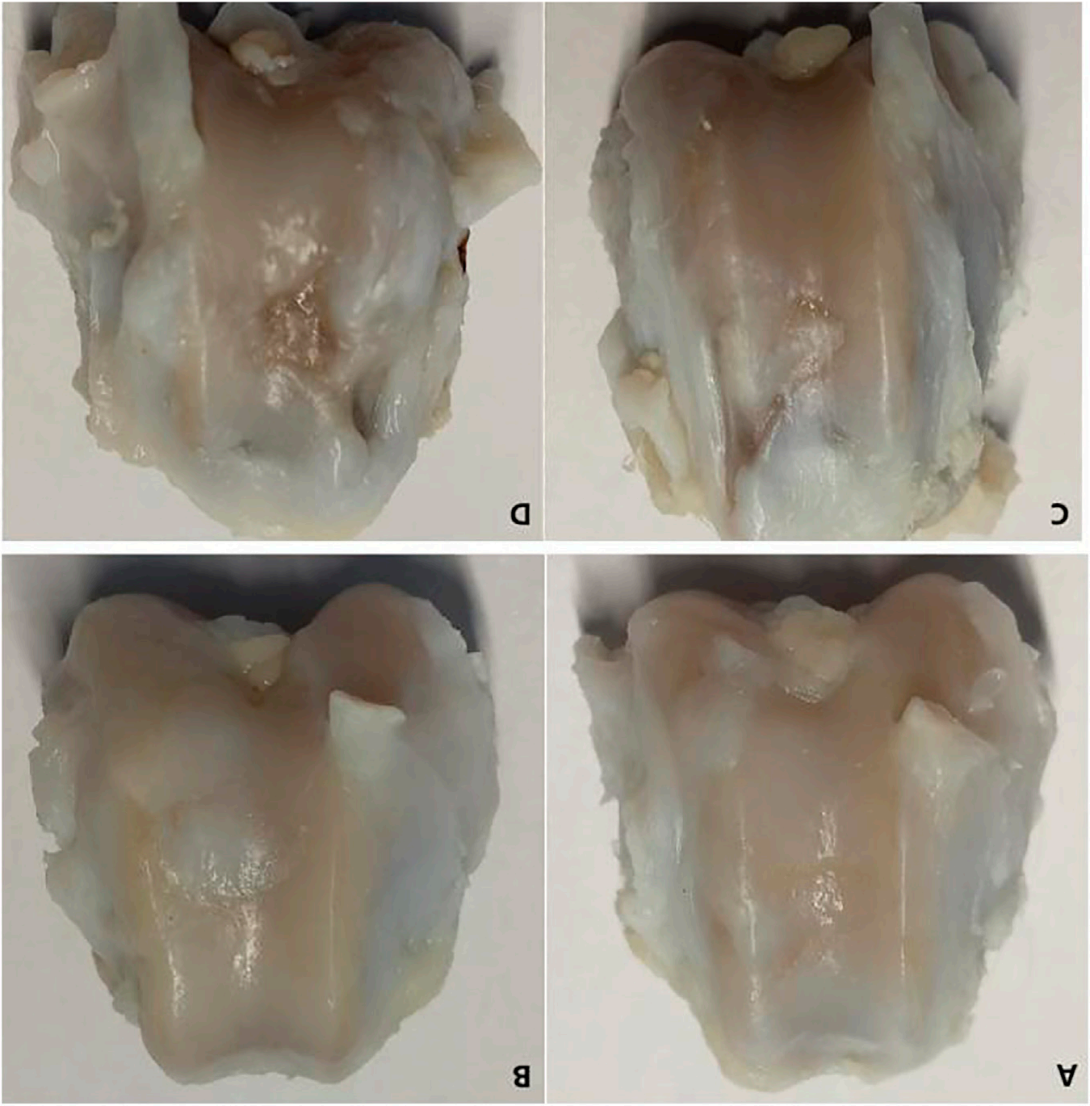
Gross views of four groups of specimens taken at 12 weeks after surgery.

The gross observation results of the joint specimen 12 weeks after surgery are shown in Figure 8. Group A: the new tissue in the cartilage defect area was filled with the relatively smooth surface and fuzzy boundary (Figure 8A). Group B: the original cartilage defect area was filled with new tissue, which was smooth and with fuzzy boundary (Figure 8B). Group C: there was new tissue in the cartilage defect area. The experimental study on the application of patellar fat pad-derived stem cells to cartilage repair revealed that the new tissue was thinner than those in groups A and B, with uneven surface and clear depression boundary (Figure 8C). Group D: the defect area was depressed, with clear boundaries, and peripheral scar-like new tissue formation (Figure 8D).

### 3.7 Comparison of Rabbit Joint Scores

**FIGURE 9:**
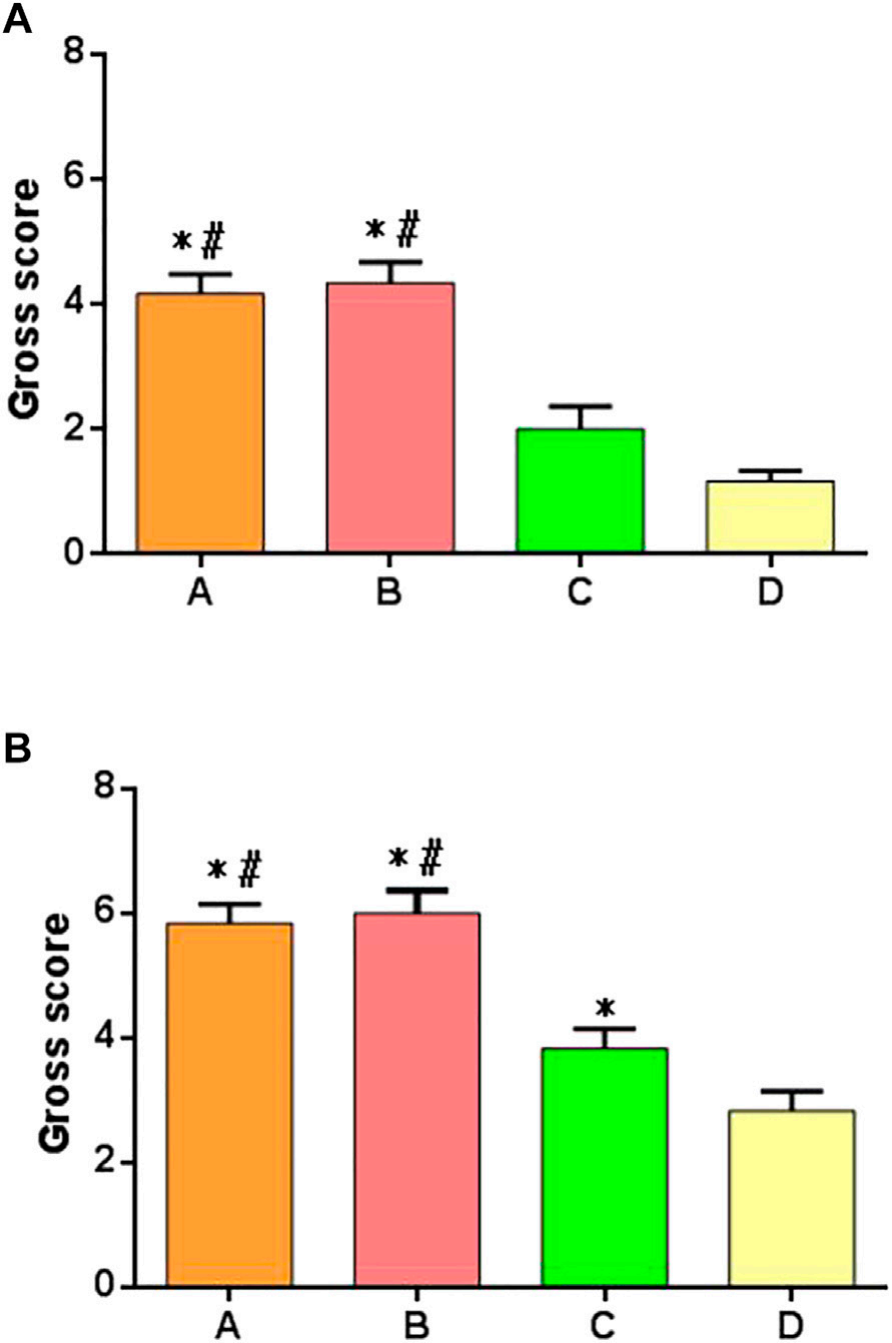
Gross joint scores of postoperative specimens in each group. **(A)** Specimen score of each group at 6 weeks after surgery. **(B)** Specimen score of each group at 12 weeks after surgery. *: significant difference compared with group D; ^#^: there was a significant difference compared with group C (*p* < 0.05).

The samples of each group were scored at 6 and 12 weeks after operation, according to the general scoring criteria of Moran’s articular cartilage. There were significant differences in the score results among multiple groups at 6 weeks after operation. There were significant differences between groups A and B compared with groups C and D, but there was no difference between groups A and B, and there was no difference between groups C and D, as shown in the Figure 9A. At 12 weeks after surgery, there were significant differences in scores among multiple groups, especially in group A or group B compared with groups C and D; there was a difference between group C and group D; there was no difference between group A and group B, as shown in Figure 9B.

### 3.8 Histological Observation of Rabbit Cartilage

**FIGURE 10:**
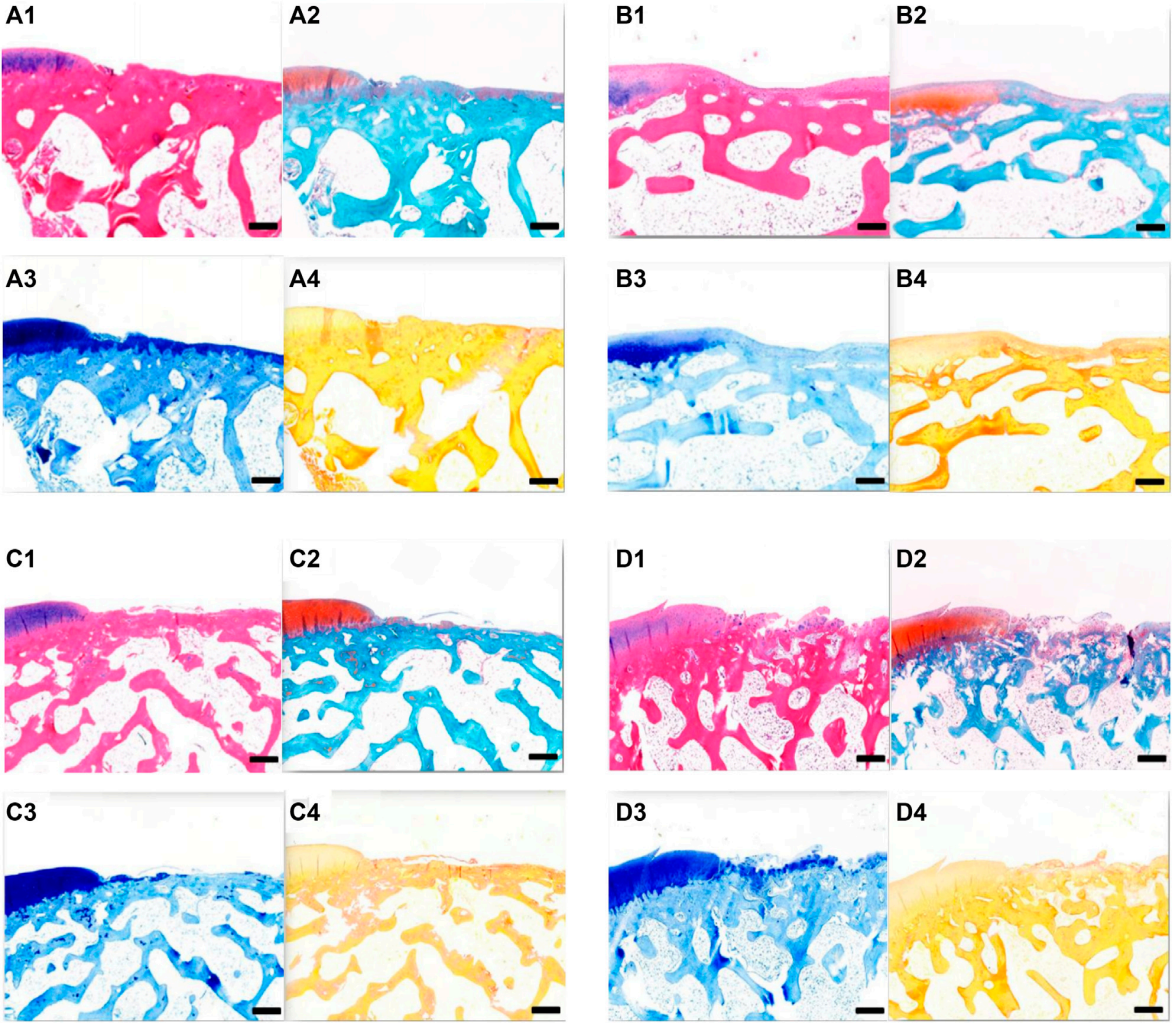
**(A)** Tissue section of group A at 6 weeks after operation. **(A1)** HE staining; **(A2)** Safranin-O/fast green staining; **A3**. Toluidine blue dyeing; **A4**. Sirius red staining. Scale = 1 mm. **(B)** Tissue section of group B at 6 weeks after operation. **B1** HE staining; **B2** Safranin-O/fast green staining; **B3** toluidine blue dyeing; **B4** Sirius red staining. Scale = 1 mm. **(C)** Tissue section of group C at 6 weeks after operation. **C1** HE staining; **C2** Safranin-O/fast green staining; **C3** toluidine blue dyeing; and **C4** Sirius red staining. Scale = 1 mm. **(D)** Tissue section of group D at 6 weeks after operation. **D1** HE staining; **D2** Safranin-O/fast green staining; **D3** toluidine blue dyeing; and **D4** Sirius red staining. Scale = 1 mm.

Cartilage repair was observed histologically 6 weeks after surgery (Figures 10A–D). In groups A and B, the bottoms of the original cartilage defect areas were covered with new cartilage layers, which were relatively continuous but uneven. In group C, the bottom of the defect area was covered with a small amount of new tissue, which was discontinuous. No significant chondrocyte generation was observed, and the concave area was not significantly filled. In group D, no new tissue was formed in the defect area and no destruction of subchondral bone was observed. All the groups had obvious boundaries between the defect area and the surrounding cartilage tissues. In groups A and B, new cartilage tissues were observed, and the new cartilage was mainly fibrocartilage, and no typical hyaline cartilage was seen.

**FIGURE 11:**
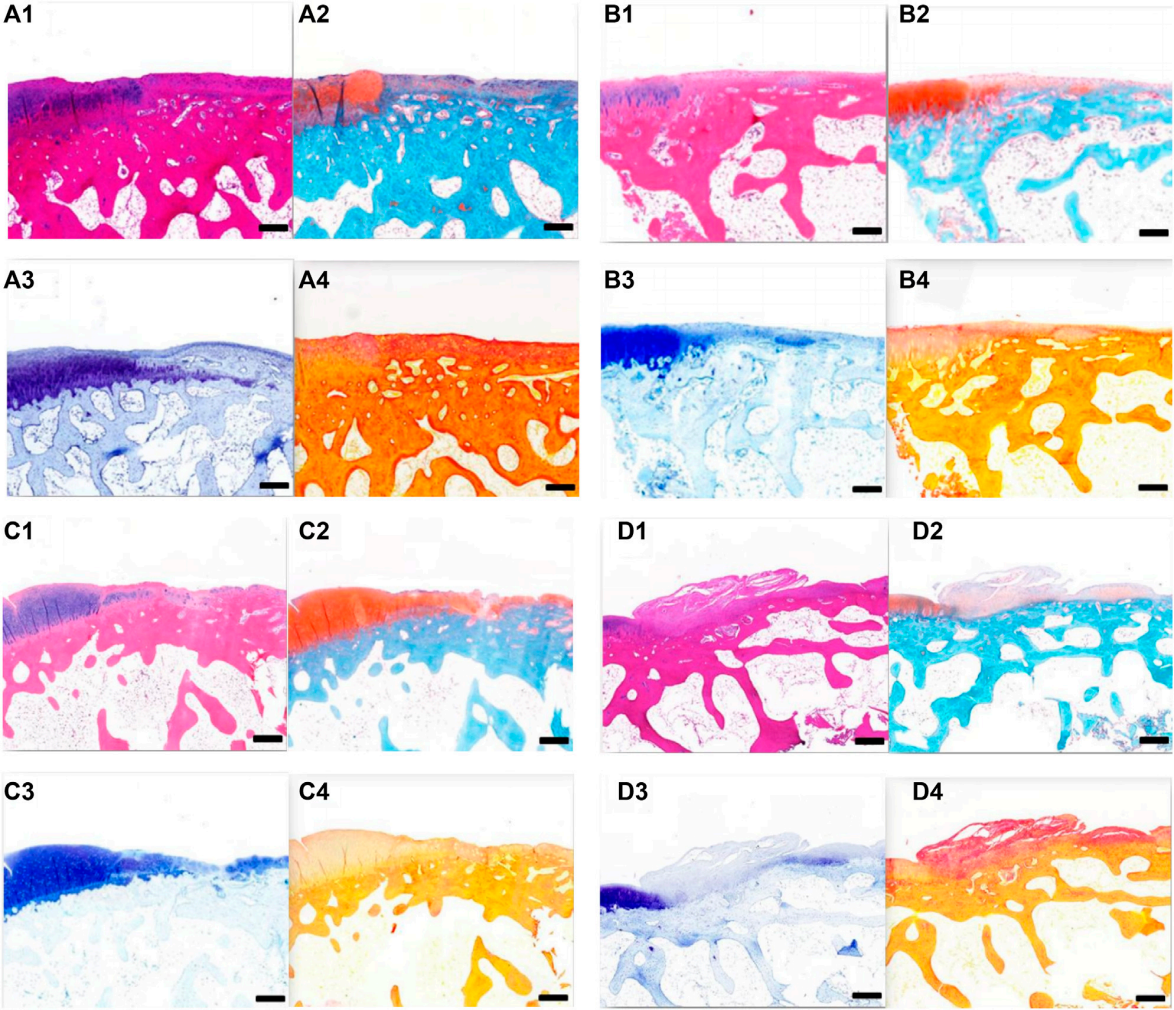
**(A)** Tissue section of group A at 12 weeks after operation. **A1** HE staining; **A2** Safranin-O/fast green staining; **A3** toluidine blue dyeing; and **A4** Sirius red staining. Scale = 1 mm. **B** Tissue section of group B at 12 weeks after operation. **B1** HE staining; **B2** Safranin-O/fast green staining; **B3** toluidine blue dyeing; and **B4** Sirius red staining. Scale = 1 mm. **C** Tissue section of group C at 12 weeks after operation. **C1** HE staining; **C2** Safranin-O/fast green staining; **C3** toluidine blue dyeing; and **C4** Sirius red staining. Scale = 1 mm. **D** Tissue section of group D at 12 weeks after operation. **D1** HE staining; **D2** Safranin-O/fast green staining; **D3** toluidine blue dyeing; and **D4** Sirius red staining. Scale = 1 mm.

Histological observation of cartilage repair at 12 weeks after surgery (Figures 11A–D) revealed that, over time, the repair effects in groups A, B, and C were better than those at 6 weeks. In both groups A and B, the defect areas were completely filled with new tissues, with a smooth surface. The new tissues were completely connected with the surrounding cartilage tissues, and the new tissues were mainly fibrocartilage. In group C, the bottom of the defect area was covered with a new layer of cartilage, which was also fibrocartilage, with a hollow and uneven surface. In group D, the surface was uneven, and fibrous connective tissue proliferation and irregularity could be seen. At 12 weeks after surgery, groups A and B had the best effect on cartilage repair, followed by group C, and group D had the worst effect.

### 3.9 Comparison of Rabbit Cartilage Tissue Scores

**FIGURE 12:**
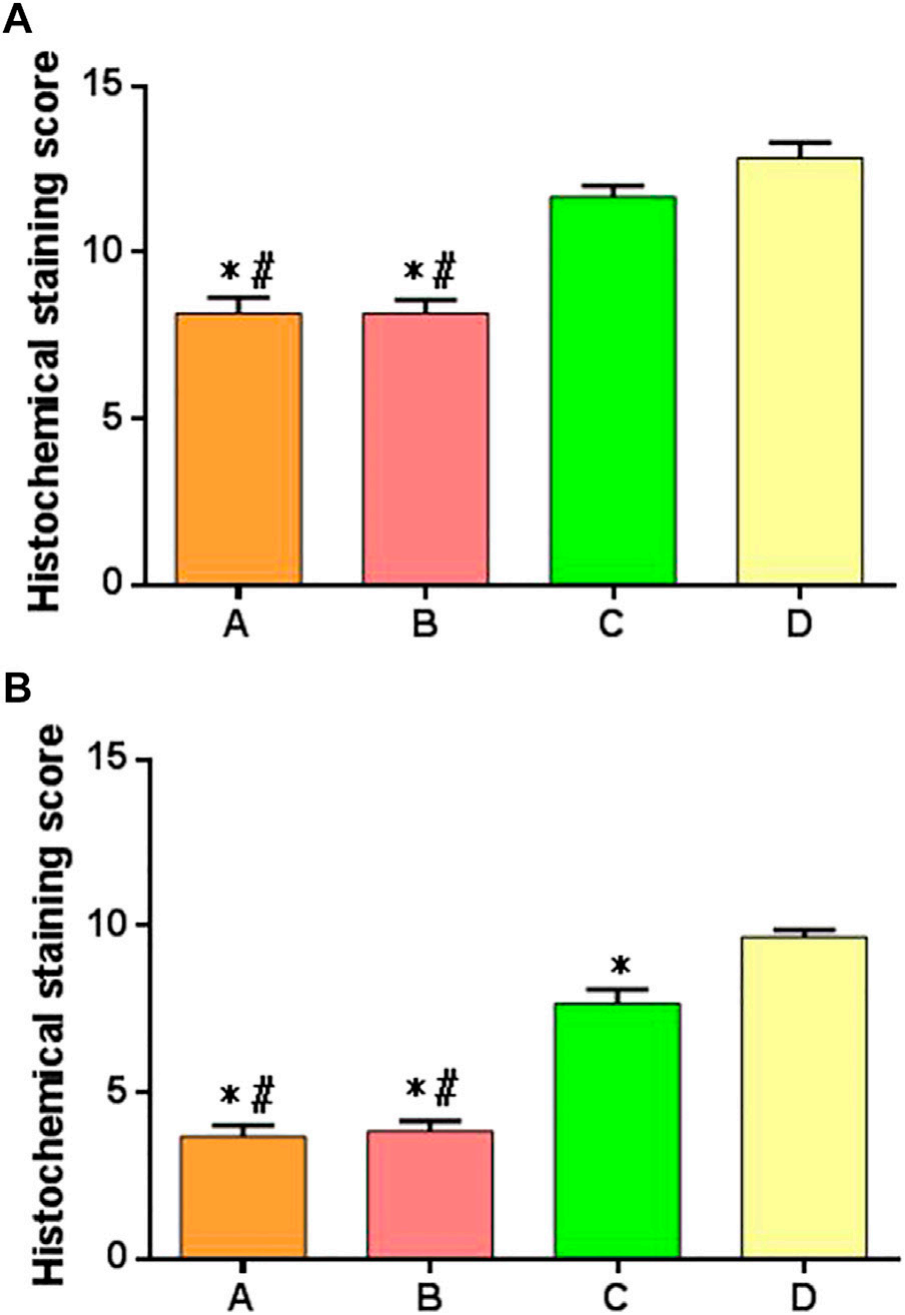
Histologic scores of cartilage repair of postoperative specimens in each group. **(A)** Specimen score of each group at 6 weeks after surgery; **(B)** specimen score of each group at 12 weeks after surgery. *: significant difference compared with group D; ^#^: there was a significant difference when compared with group C (*p* < 0.05).

The histological scoring criteria of cartilage repair were used to score each group of specimens at 6 and 12 weeks after surgery. There were significant differences in the score results among multiple groups at 6 weeks after operation. There were significant differences between groups A or B compared with groups C and D, but there was no difference between groups A and B, and there was no difference between groups C and D, as shown in Figure 12A. At 12 weeks after operation, there was significant difference in the scores among multiple groups, especially in the group A or group B compared with the groups C and D; there was a difference between the group C and group D; there was no difference between the group A and group B, as shown in Figure 12B.

## 4 Discussion

The method of rapid, effective, and safe isolating of large amounts of ADSCs from adipose tissue is of great significance and is also of great clinical and market demand. The clinical approaches to obtain SVF using enzyme digestion or the principle of the instrumentation used are based on enzyme digestion in the laboratory for the isolation of ADSCs, and there is no certain standard for the composition and concentration of collagenase used in the method, and different isolation methods may result in different cell yields.

In this study, we obtained IPFP-SCs from rabbit infrapatellar fat pad tissue by without enzyme isolation. This method is simple to operate, low in cost, and does not require the use of expensive equipment and collagenase. In the primary extraction test of the two kinds of cells, the total number of cells by enzyme digestion method was higher than that by the without enzyme method. However, in this part of the experimental study, IPFP-SCs obtained by without enzyme isolation could adhere and grow after seeding in polystyrene culture flasks and showed the characteristics of clustered growth, and the flow cytometry (FC) assay suggested: CD44^+^, CD90^+^, CD34^−^, and CD45^−^. In the three-way differentiation potential experiment, the cells after osteogenic-, adipogenic-, and chondrogenic-induced differentiation all showed positive results after corresponding staining treatments, in accordance with the basic characteristics of MSCs (Bourin et al., 2013).

Report of DSCs obtained by the without enzyme method suggest that the yield of nucleated cells is significantly diminished, with the recovered cell population containing more peripheral blood mononuclear cells and a significantly reduced number of progenitor cells. This is because ADSCs are mainly concentrated around small- and medium-sized vascular structures in adipose tissue, and in the absence of enzymatic digestion to break down ECM, many stem cells remain in the endothelial layer of blood vessels and connective tissue fragments. Although the enzymatic digestion method has higher yields and contains more progenitor cell components, the mechanics without enzyme isolation method does show significant advantages. The digestive enzymes required for adipose tissue digestion are expensive, and in addition, the without enzyme method can provide faster processing time, with the acquisition of ADSCs typically taking less than 15 min and not requiring an additional 30 min to 2 h for the enzymatic digestion process (Aronowitz and Ellenhorn, 2013; Raposio et al., 2017).

Articular cartilage is a hyaline cartilage, which is neither innervated nor vascularized and has a very low cell density, resulting in cartilage with a very limited ability to self-repair (Jevotovsky et al., 2018). With the continuous development of regenerative medicine, it brings hope for the regenerative repair of cartilage. However, how to implement stem cells safely and effectively in the clinic is also something that needs to go into depth. We designed the animal model of cartilage defect in rabbits and chose rabbit subpatellar fat pad tissue-derived stem cells for the selection of stem cells. It was suggested that IPFP-SCs of OA knee joints and healthy knees (patients undergoing cruciate ligament reconstruction surgery) origin, showed similar chondrogenic potential, which also provided a powerful basis for our experimental application of IPFP-SCs (Liu et al., 2014).

The results of the study showed that the use of IPFP-SCs of either by the without enzyme method or enzyme digestion method had a significant repair-promoting effect compared to the blank group using normal saline. In particular, the repair effect on cartilage was clearer after the combined use of P-PRP. IPFP-SCs exhibit unique properties in terms of cell proliferative capacity, multidirectional differentiation potential, and available tissue reserves (Mochizuki et al., 2006; Pires de Carvalho et al., 2014). From the point of view of embryonic origin, the potential of stem cells from the inferior fat pad tissue for the repairing articular cartilage is unique and more significant compared to other sources of stem cells. It has been hypothesized that a large number of stem cells and pericytes reside in the subpatellar adipose tissue, and when the joint is damaged, these stem cells or pericytes migrate into the synovial lining and into the synovial fluid in an attempt to reach and repair the damaged articular cartilage (Zimmerlin et al., 2013). It would be beneficial to translate laboratory-developed techniques to the clinical setting if they could be performed quickly, aseptically, safely, in a single step, or if they could be performed in a day room. The application protocols of stem cells also need to be continuously optimized in order to obtain better clinical applications and clinical outcomes.

However, there are other limitations to the current study. First, the sample size is small, and the follow-up period is short. In addition, only P-PRP and IPFP-SCs were used to repair articular cartilage, but there was no corresponding control group. Therefore, more animal studies and a relatively long observation period are needed in the future. Second, the potential molecular mechanisms of cartilage repair need further investigation.

In summary, obtaining IPFP-SCs by the without enzyme method was less time-consuming than that with the enzyme digestion method, and there was no significant difference in cell identification and differentiation potential between the two methods. However, the rate of obtaining primary cells was significantly lower than that with the enzyme digestion method. IPFP-SCs showed good repair effect in the rabbit cartilage defect model and provided ideas and reference for the clinical application of stem cells in the repair of articular cartilage.

## Data Availability

The original contributions presented in the study are included in the article/Supplementary Material; further inquiries can be directed to the corresponding authors.
